# XDR-TB in South Africa: Detention Is Not the Priority

**DOI:** 10.1371/journal.pmed.0040162

**Published:** 2007-04-24

**Authors:** Eric Goemaere, Nathan Ford, Daniel Berman, Cheryl McDermid, Rachel Cohen

We agree that there is “no time for denial or complacency” when it comes to the spread of MDR- and XDR-TB in South Africa. Unfortunately, the attention the recent *PLoS Medicine* article [1] generated in South Africa and internationally has overwhelmingly focused on detention of patients. Headlines such as “South Africa urged to isolate ‘killer’ TB patients” [2] place the blame on patients and divert attention from more urgent priorities.

The TB epidemic in South Africa, as across sub-Saharan Africa, is largely linked to HIV. In Khayelitsha Township near Cape Town, new cases had risen to around 2,000 per 100,000 in 2006, fuelled by the high prevalence of HIV. From our experience in South Africa, a number of challenges must be addressed locally and nationally to curb MDR-TB. Detention does not come high on this list.

Effective MDR-TB management requires improvements in general TB control, but this alone will not remove the need to respond to MDR-TB. The Western Cape has the best TB outcomes in South Africa, thanks to enormous investments in TB control, but despite this MDR- and XDR-TB cases are increasingly being reported. There is an urgent priority for infection control, taking into account the context of limited resources at the primary care level and high HIV prevalence. Data from mid-2006 show that 67% of TB patients in Khayelitsha are HIV positive; in Médecins Sans Frontières' programme in Lesotho the figure rises to 92%. Patient triage is one aspect, but the reality is that undiagnosed MDR- and XDR-TB patients with HIV are sitting in overcrowded waiting rooms next to other immunocompromised patients. Personal protection for health staff, starting with basic training on infection control, needs to be improved. Structural improvements to the clinics need to be based on feasible, low-tech solutions—air extractors and windows will be more practical than UV lights and negative pressure rooms [3].

Access to points of care needs to increase. The Western Cape has reported over 800 cases of MDR-TB in the last two years and this is certainly an underestimation. Greater diagnostic capacity and more rapid diagnosis is needed, and diagnosis must be met with better access to treatment. Treating MDR-TB currently relies on hospitalization of patients, but current needs are far greater than hospital capacity—patients can wait up to four months for a hospital bed. The traditional model of leaving MDR-TB management to specialists has incapacitated health-care staff at the primary care level who receive little or no training on how to manage MDR-TB. In other settings in southern Africa the situation is even worse. In Lesotho there is practically no access to reliable culture or drug-sensitivity testing. Given the scarcity of human resources and the overwhelming number of co-infected patients, treatment needs to be delivered in as decentralized a manner as possible.

In settings where clinics are saturated and patient numbers are rising, it is not realistic to rely on a strategy of simply reinforcing directly observed treatment and incarcerating defaulters to respond to MDR-TB. We need to apply the lessons learnt from providing HIV care in resource-poor settings, including decentralization of services to the primary care level, reinforcing adherence through treatment literacy and a patient-centred approach, and community-based support. The reality, though, is that an integrated approach to HIV and TB is far away: around a third of MDR-TB patients in Khayelitsha do not even know their HIV status.

Drug-resistant TB is not a new problem. What is new is the willingness to detect and treat it. The lack of willingness to do so until recently has left us with old drugs and diagnostics that make treating drug-resistant TB at best highly complex and resource intensive, and at worst impossible. Programme-level improvements have to be met with a dramatic increase in efforts to develop new drugs and diagnostics [4].
